# Diagnosis of Parasitic Diseases: Old and New Approaches

**DOI:** 10.1155/2009/278246

**Published:** 2009-12-30

**Authors:** Momar Ndao

**Affiliations:** National Reference Centre for Parasitology, McGill University Centre for Tropical Diseases, Montreal General Hospital, 1650 Cedar Avenue R3-137, Montreal, QC, Canada H3G 1A4

## Abstract

Methods for the diagnosis of infectious diseases have stagnated in the last 20–30 years. Few major advances in clinical diagnostic testing have been made since the introduction of PCR, although new technologies are being investigated. Many tests that form the backbone of the “modern” microbiology laboratory are based on very old and labour-intensive technologies such as microscopy for malaria. Pressing needs include more rapid tests without sacrificing sensitivity, value-added tests, and point-of-care tests for both high- and low-resource settings. In recent years, research has been focused on alternative methods to improve the diagnosis of parasitic diseases. These include immunoassays, molecular-based approaches, and proteomics using mass spectrometry platforms technology. This review summarizes the progress in new approaches in parasite diagnosis and discusses some of the merits and disadvantages of these tests.

## 1. Introduction

Currently, the detection and diagnosis of parasite infections rely on several laboratory methods in addition to clinical symptoms, clinical history, travel history, and geographic location of patient. The primary tests currently used to diagnose many parasitic diseases have changed little since the development of the microscope in the 15th century by Antonie van Leeuwenhoek. Furthermore, most of the current tests cannot distinguish between past, latent, acute, and reactivated infections and are not useful for following response to therapy or for prognosis.

 Recent developments in new diagnostic tools, however, have opened new avenues for a vast improvement in parasite detection. Firstly, a number of newer serology-based assays that are highly specific and sensitive have emerged, such as the Falcon assay screening test ELISA (FAST-ELISA) [1], Dot-ELISA [2, 3], rapid antigen detection system (RDTS) [4], and luciferase immunoprecipitation system (LIPS) [5]. Secondly, molecular-based approaches such as loop-mediated isothermal amplification (LAMP) [6], real-time polymerase chain reaction [7], and Luminex [8] have shown a high potential for use in parasite diagnosis with increased specificity and sensitivity. Thirdly, proteomic technology has also been introduced for the discovery of biomarkers using tissues or biological fluids from the infected host.

 The aim of this review is to highlight the potential for these new technologies in parasite diagnosis. For convenience, old and new parasitic diagnostic tools are summarized in Tables 1 and 2. The diagnostic tools offered by the CDC (Centre for Disease Control, Atlanta, USA) and the NRCP (National Reference Centre for Parasitology, Montréal, Canada) are also highlighted in Tables 3 and 4.

## 2. Microscopy

For many years, microscopy has been the only tool available for the detection of parasites through inspection of blood smears [10–14], tissue specimens [15–17], feces, lymph node aspirates [18, 19], bone marrow [20], and even cerebrospinal fluid [21]. However, sample preparation for direct observation is time-consuming, labour intensive, and proper diagnosis depends on qualified laboratory technicians. In the case of slide reading, a second independent reading is preferable, but not always required for accurate diagnosis. If need be, divided readings are resolved by a third reader. In endemic regions, where resources are limited, this proves to be difficult and misdiagnosis can significantly impact patient care. In reality, all major intestinal helminth infections are still solely dependent on microscopy for diagnosis. As for other parasite infections, many are confirmed by the use of microscopy in conjunction to other methods of diagnosis including serology-based assays and more recently molecular-based assays.

## 3. Serology-Based Assays

In situations where biologic samples or tissue specimens are unavailable, serology alone is the gold standard for diagnosis. Serology-based diagnosis tools can be divided into two categories: antigen-detection assays and antibody-detection assays. These include the enzyme-linked immunosorbent assay (ELISA), also called enzyme immunoassay (EIA), and all its derived tests such as the Falcon assay screening test ELISA (FAST-ELISA) and the dot-ELISA. Other assays include the hemagglutination (HA) test, indirect or direct immunofluorescent antibody (IFA or DFA) tests, complement fixation (CF) test, and immunoblotting and rapid diagnostic tests (RDTs). 

 Although the ease of use and turnaround times for serologic assays are similar to microscopy, serology-based assays are more sensitive and specific. It becomes important for individuals whose blood smears do not permit identification of the parasite (e.g., differentiating between *Babesia* and *Plasmodium*) [159] or for patients exhibiting low-parasitemia and/or who are asymptomatic (e.g., Chagasic patients) [54]. Classifying an infected asymptomatic patient as negative could lead to transmission of the parasite during blood transfusions or organ transplants. In the case of *Fasciola* infection, serology tests have also been shown to be useful in the confirmation of chronic fascioliasis when egg production is low or sporadic [112]. Finally, having these tests readily available allows for the monitoring of parasite clearance following therapy. 

### 3.1. Falcon Assay Screening Test ELISA (FAST-ELISA)

The Falcon assay screening test ELISA (FAST-ELISA) consists of using synthetic and recombinant peptides to evaluate antibody responses to an antigen [1]. In the past, the method has been applied to the study of malaria [32], fasciolosis [112], schistosomiasis (reviewed in [160]), and taeniasis [161]. However, this technique is subjected to the same drawbacks as most serology-based tests. Antibodies raised against a peptide from one parasite protein may cross-react with proteins from other species. Moreover, antibodies raised against a peptide may react in some assays but not in others and some regions of a peptide may be more immunogenic than others. No recent studies have been published on the use of the FAST-ELISA for the diagnosis of parasitic infections.

### 3.2. Dot-ELISA

The main difference between the regular ELISA and the dot-ELISA lies in the surface used to bind the antigen of choice. In the dot-ELISA, the plastic plate is replaced by a nitrocellulose or other paper membrane onto which a small amount of sample volume is applied. The choice of binding matrix greatly improved the specificity and sensitivity of the assay by reducing the binding of nonspecific proteins usually observed when plastic binding matrixes are used. The principle is similar to the immunoblot. The dotted membrane is incubated first with an antigen-specific antibody followed by an enzyme-conjugated anti-antibody. The addition of a precipitable, chromogenic substrate causes the formation of a colored dot on the membrane which can be visually read [2]. The benefits of this technique include its ease of use, its rapidity, and the ease of result interpretation. It is fast, and cost-effective and more importantly can be used in the field (e.g., as a dipstick). For all these reasons, the Dot-ELISA has been and still is extensively used in the detection of human and animal parasitic diseases, including amebiasis, babesiosis, fascioliasis, cutaneous and visceral leishmaniasis, cysticercosis, echinococcosis, malaria, schistosomiasis, toxocariasis, toxoplasmosis, trichinosis, and trypanosomiasis (all reviewed in [3]). In the last few years, published studies have demonstrated the use of the dot-ELISA for the detection of *Fasciola gigantica* [113], *Haemonchus contortus* [162], *Theileria equi* [163], *Trypanosoma cruzi* [164], and *Trypanosoma brucei* [34]. In the latter study the researchers were able to demonstrate that the dot-ELISA had better sensitivity and specificity than the ELISA in the detection of antineurofilament and antigalactocerebrosides antibodies in cerebrospinal fluid of subjects infected with African trypanosomes. They attributed the greater sensitivity and specificity of the dot-ELISA to the use of the nitrocellulose membrane and showed that their assay was successfully reproducible in the field.

### 3.3. Rapid Antigen Detection System (RDTS)

Rapid antigen detection tests (RDTs) based on immunochromatographic antigen detection have been implemented in many diagnostic laboratories as an adjunct to microscopy for the diagnosis of malaria. RDTs consist of capturing soluble proteins by complexing them with capture antibodies embedded on a nitrocellulose strip. A drop of blood sample is applied to the strip and eluted from the nitrocellulose strip by the addition of a few drops of buffer containing a labeled antibody. The antigen-antibody complex can then be visualized directly from the membrane [4].

 Since the appearance of the first RDTs in the 990s, major improvements have been made to the technique, making the use of RDTs in rural endemic regions feasible. RDTs are now rapid, stable at temperatures up to 40°C, easy to use, and cost-effective thereby providing many advantages over traditional microscopic methods [165]. RDTs are useful in the identification of *P. falciparum* and *P. vivax* infections but cannot be used to identify *P. malariae* and *P. ovale* infections [4]. In addition, they are useless at detecting very low-density infections. PCR-based approaches remain the tool of choice in that situation. More than 80 RDTs exist for the detection of either histidine-rich protein (HRP) specific to *P. falciparum* or species-specific isotypes of lactate dehydrogenase (LDH) [49]. However, as reported by Murray et al. [165] only 23 have met the WHO's criteria for international marketing. 

 Malaria RDTs have recently been introduced in African countries to help prevent misdiagnosis of malaria infections and to subsequently reduce the practice of presumptive treatment [49]. In fact, the tendency to treat slide-negative samples with antimalarials is still a common phenomenon. This practice causes concern not only for the patient's health care but also to the costs it generates in prescribing the more expensive antimalarial sulfadoxine/pyrimethamine and artemisinin-based combinations [165]. Finally, misuse of antimalarials could lead to the appearance of drug-resistant strains.

### 3.4. Luciferase Immunoprecipitation System (LIPS)

The luciferase immunoprecipitation system (LIPS) is a modified ELISA-based assay in which serum containing antigen-specific antibodies can be identified by measuring light production. Basically, an antigen of choice is fused to the enzyme reporter Renilla luciferase (Ruc) and expressed as a Ruc-fusion in mammalian cells to allow for mammalian-specific posttranslational modifications. The crude protein extract is then incubated with the test serum and protein A/G beads. During the incubation, the Ruc-antigen fusion becomes immobilized on the A/G beads, which allows the antigen-specific antibody to be quantitated by washing the beads and adding coelenterazine substrate and measuring light production [5]. 

 In recent years, LIPS has been successfully applied for the identification of sera samples infected with *Strongyloides stercoralis* (using a Ruc-NIE fusion) [134] and *Loa loa* (using a Ruc-LlSXP-1 fusion) [133]. Some of the advantages of the LIPS technology include its rapidity and accuracy in detecting infected patients. Sensitivity is improved in part by the use of mammalian cells which produce fusion antigens free of contaminating bacterial proteins. In addition, low backgrounds are produced compared to the ELISA. This greatly facilitates the separation between negative and positive samples. In addition, the *Strongyloides* LIPS based on the NIE antigen showed greater specificity than the ELISA as no cross-reaction was observed with serum from filarial-infected subjects [134].

 A LIPS assay can be performed in 2.5 hours. Burbelo et al. 2008 [133] were able to obtain 100% specificity and sensitivity when performing an LIPS assay based on the *Loa loa* SXP-1 antigen with only a small-degree of cross-reactivity with a few *Onchocerca volvulus*- and *Wuchereria bancrofti*-infected patient sera. By decreasing the incubation times of a normal LIPS assay, they were able to minimize cross-reaction. Many of the *O. volvulus* sera samples tested as positive with the LIPS assay were negative using this 15-minute LIPS assay also called QLIPS. Of interest for the application of this technique in the field is the observation that blood obtained by finger-prick (contaminated with red blood cells and other components) did not interfere with the LIPS assay. Further studies will be useful in exploring and validating the accuracy and potential usefulness of the LIPS and QLIPS assays in the field. 

 As discussed, immunodiagnostics tests have some serious limitations. Parasitic diseases such as amebiasis, cryptosporidiosis, filariasis, giardiasis, malaria, cysticercosis, schistosomiasis, and African trypanosomiasis do not have commercially or FDA approved antibody detection tests for their diagnosis. Experimental results have been too variable due to the type of antigen preparations used (e.g., crude, recombinant purified, adult worm, egg) and also because of the use of nonstandardized test procedures. Cross-reaction leading to false-positives and misdiagnosis is also a problem, especially in regions where more than one parasite is endemic. Despite the fact that some parasites in South America share common epitopes, it is common to see coinfection with *Trypanosoma cruzi* and *Leishmania* species [166]. It is also a problem in Africa, where cross-reactivity exists between filarial and other helminth antigens [133]. To a lesser extent but nonetheless important is the inability of antibody-detection tests to differentiate between past and currently active infections [167]. Furthermore, antibody-detection tests cannot be used in parasitic infections that do not develop a significant antibody response. This has been observed in some individuals carrying *Echinococcus* cysts [168] or during cutaneous leishmaniasis (http://www.dpd.cdc.gov/dpdx/HTML/Leishmaniasis.htm). Similarly, in the case of African trypanosomiasis diagnosis, such tests are of limited use because seroconversion occurs only after the onset of clinical symptoms [83].

 For all these reasons, there is still a need to improve on the current diagnosis approaches available. Since the advent of the polymerase chain reaction (PCR), parasitologists have turned to molecular-based approaches in the hopes to better the existing diagnosis tools.

## 4. Molecular-Based Approaches

### 4.1. Nucleic Acid-Based Approaches

The many limitations of microscopy and serology-based assays have influenced parasitologists towards the use of gene amplification methods made possible with the advent of the polymerase chain reaction (PCR). Besides the traditional PCR, including nested and multiplexed PCR, we have seen the implementation of the real-time PCR (RT-PCR) for the detection of several parasitic infections. Newer technologies such as loop-mediated isothermal amplification and Luminex-based assays have also emerged as possible new approaches for the diagnosis of parasitic diseases.

 Molecular-based approaches based on nucleic acids offer greater sensitivity and specificity over the existing diagnostic tests. They permit the detection of infections from very low parasitized samples including those from asymptomatic patient's samples [169]. Moreover, multiplexed PCR allows for the detection of multiple sequences in the same reaction tube proving useful in the diagnosis of several parasitic infections simultaneously [170].

### 4.2. Real-Time Polymerase Chain Reaction (RT-PCR)

RT-PCR system unlike conventional PCR, allow for the quantification of the original template's concentration through the use of various fluorescent chemistries, such as Sybergreen, Taqman probes, fluorescence resonance energy transfer (FRET), and Scorpion primers [7]. The concentration is measured through comparison to standard curves. This eliminates the need to visualize the amplicons by gel electrophoresis thereby greatly reducing the risk of contamination and the introduction of false-positives. When multiplexed, RT-PCR allows for the high-throughput analysis of different sequences in one single-closed tube reaction [171]. Using multiplexed RT-PCR, Shokoples et al. [4] were able to identify the four human *Plasmodium* species (*falciparum*, *vivax*, *malariae*, and *ovale*) in a single reaction tube even in very low parasitized samples. Running the multiplex assay not only reduced the cost per test but also allowed for a rapid turnaround time, the assay taking only three hours to complete. It is a clear advantage over microscopy which is labour intensive and time-consuming with slow turnaround times especially during high-throughput settings. Similarly, multiplexed RT-PCR proved useful in differentiating drug-sensitive strains of malaria [172]. This is important for proper antimalarial prescription. In another example, Diez et al. [54] were able to detect the presence of *T. cruzi* infection following heart transplants using PCR. This allowed immediate treatment of the patients before reactivation of Chagas disease could occur. These examples demonstrate that efficient and early diagnosis can directly impact patients care and that PCR-based approaches have the potential to help in making the right choice for treatment. 

 Although DNA-based methods have shown excellent sensitivity and specificity, the introduction of these methods in daily laboratory practice is still uncommon especially in rural endemic regions. In addition, as observed with many serology-based assays, PCR-based methods also suffer by the lack of standardization [22]. DNA extraction, choice of primer sets, and use of various amplification protocols are all factors that may cause this diversification in results [173]. Adding an automated DNA extraction step would certainly improve PCR assays for use in the diagnosis of parasitic diseases.

### 4.3. Loop-Mediated Isothermal Amplification (LAMP)

Loop-mediated isothermal amplification (LAMP) is a unique amplification method with extremely high specificity and sensitivity able to discriminate between a single nucleotide difference [6]. It is characterised by the use of six different primers specifically designed to recognise eight distinct regions on a target gene, with amplification only occurring if all primers bind and form a product [174]. In the past, LAMP has been successfully applied for the rapid detection of both DNA and RNA viruses such as the West Nile [175] and SARS viruses [176]. Recently, parasitologists have adapted the LAMP approach for the detection of several parasitic diseases including the human parasites *Entamoeba* [177], *Trypanosoma* [68], *Taenia* [153], *Plasmodium* [70], and *Cryptosporidium* [152], the animal parasites *Theilera* [178] and *Babesia* [178, 179], and even to the identification of vector mosquitoes carrying *Plasmodium* [73] and *Dirofilaria immitis* [180] parasites. Most of these studies have brought to light the many advantages of this method over the common PCR technique.

 Unlike a regular PCR reaction, LAMP is carried out at a constant temperature (usually in the range of 60–65°C). This unique feature not only results in higher yields, but also eliminates the need to buy a thermal cycler and shortens the reaction time by eliminating time lost during thermal changes. In addition, the reaction can be carried out without extracting the DNA from the collected samples as shown in the case of RIME, a nonautonomous retroelement found in *Trypanosoma brucei rhodesiense* and *T. b. gambiense* [68]. In 35 minutes, using a simple water bath, RIME LAMP was able to detect both *T. b. gambiense* and *T. b. rhodesiense* directly from blood, serum, and CSF samples. More importantly, the study has shown reproducibility in the field. In addition to the above advantages, LAMP reactions are easy to set up, and results can readily be assessed. The sample of interest is mixed with primers, substrates, and a DNA polymerase capable of strand displacement in a microcentrifuge tube. During the reaction, large amounts of pyrophosphate ions are produced, leading to the formation of a white precipitate [181]. This turbidity is proportional with the amount of DNA synthesized therefore one can assess the reaction by real-time measurement of turbidity or more importantly, simply through the naked-eye. 

 For all these reasons, the future adoption of LAMP as a diagnostic tool for parasite infections in rural endemic regions shows promise. Furthermore, as more groups apply LAMP to the field of parasitology, we will see the appearance of LAMP-modified assays that meet specific detection needs. For example, in a recent study on bovine Babesia [182], a multiplex-LAMP (mLAMP) assay was developed to simultaneously detect *B. bovis* and *B. bigemina* from DNA extracted from blood spotted on filter paper. Similarly, Han et al. [71] implemented a LAMP assay based on the 18S rRNA gene for the detection of the four human *Plasmodium* species (*falciparum*, *vivax*, *malariae*, and *ovale*). LAMP had a similar sensitivity and a greater specificity than nested PCR, yielding similar results but at a faster turnaround time. Their results are consistent with other studies demonstrating the rapidity and the improved specificity and sensitivity obtained using the LAMP assay.

### 4.4. Luminex xMAP Technology

Luminex technology is a bead-based flow-cytometric assay that allows the detection of various targets simultaneously (http://www.luminexcorp.com/). The microsphere beads can be covalently bound to antigens, antibodies, or oligonucleotides that will serve as probes in the assay. Up to 100 microspheres are available each emitting unique fluorescent signals when excited by laser therefore allowing the identification of different targets [183]. Adapted to the study of parasites, the Luminex assay could identify multiple organisms or different genotypes of one particular organism during the same reaction utilizing very low volume. The approach could prove useful in the study of antigenic diversity and drug-resistance alleles and for the diagnosis of parasitic diseases.

 Luminex was applied to the study of *Cryptosporidium* [154]. *C*. *hominis* and *C. parvum* cannot be distinguished using antigen detection or serology assays. Only DNA-based approaches have been successful in doing so by exploiting the single nucleotide difference in the microsatellite-2 region (ML-2) of both species [154]. Ultimately DNA sequencing is the diagnosis tool of choice but it is costly, labour-intensive and time-consuming. In a recent study, Bandyopadhyay et al. [154] successfully detected and distinguished *C. hominis* and *C. parvum* in 143 DNA extracts using Luminex technology by using oligonucleotide probes specific to the ML-2 regions of each species. Turnaround time was about 5 hours making this assay not only much faster but also less expensive than PCR followed by DNA sequencing. It also proved to be 100% specific and more sensitive than a direct fluorescent antibody (DFA) test, a method routinely used to identify *Cryptosporidium* and *Giardia* species. Note that DFA cannot differentiate between *C. hominis* and *C. parvum*.

 Similarly in other research, Luminex technology was able to detect all-blood stage parasite levels of the four human *Plasmodium* species (*falciparum*, *vivax*, *malariae*, and *ovale*) simultaneously [75]. This study demonstrated that Luminex technology can improve the speed, the accuracy, and the reliability of other PCR methods. For example, the need for gel electrophoresis to differentiate the LDR products representing the four human Plasmodium species is eliminated. Second, all samples are handled simultaneously and continuously through a 96-well plate format from DNA extraction all through data analysis. The process is automated and therefore uniformity can be achieved. Finally, the high-throughput capability of the Luminex system confers it a clear advantage over the use of labour-intensive microscopy for large scale studies.

### 4.5. Proteomics

Since proteins are the main catalysts, structural elements, signalling messengers, and molecular machines of biological tissues, proteomic studies are able to provide substantial clinical relevance. Proteins can be utilized as biomarkers for tissues, cell types, developmental stages, and disease states as well as potential targets for drug discovery and interventional approaches. The next generation of diagnostic tests for infectious diseases will emerge from proteomic studies of serum and other body fluids. Recent advances in this area are attributable largely to the introduction of mass spectrometry platforms capable of screening complex biological fluids for individual protein and peptide “biomarkers.” Proteomic strategy can identify proteins in two ways: bottom-up and top-down approaches. In the former, the proteins in a biological fluid are proteolytically shattered into small fragments that can be easily sequenced and the resultant spectra are compared with those in established peptide databases. This is the protein equivalent of “shotgun” genomics. Bottom-up strategies are difficult to quantitate and cannot identify modified molecules (e.g., alternately spliced, glycosylated). Since each open reading frame in the human genome is thought to generate at least 10 modified proteins, this issue is a major limitation. 

 The classic top-down strategy is 2-dimensional gel electrophoresis. Top-down strategies seek to identify proteins and peptides (and their natural variants) in complex biological fluids. Two-dimensional (2D) gel electrophoresis was first described in 1975. With this method, proteins are resolved in the first dimension based on pH (a process called isoelectric focusing) and in the second dimension by their molecular weight. This technique is labor intensive, and low throughput and requires large amounts of sample. Such limitations have encouraged the search for improved approaches. Other techniques used for the expression analysis of proteins are matrix-assisted laser desorption ionization time-of-flight mass spectrometry (MALDI-TOF MS), surface-enhanced laser desorption ionization time of flight mass spectrometry (SELDI-TOF MS), liquid chromatography combined with MS (LC–MS–MS), isotope-coded affinity tags (ICAT), and isotope tags for relative and absolute quantification (iTRAQ).

 The development of automated, high-throughput proteomic technologies such as MALDI-TOF and SELDI-TOF MS has enabled large numbers of clinical samples to be analyzed simultaneously in a short time. These platforms have made “population-based proteomics” feasible for the first time (reviewed in [184]). All proteomics-based diagnostic efforts seek to identify biomarkers that, alone or in combination, can distinguish between “case” and “control” groups. 

 The main limitation of SELDI compared to MALDI resides in the fact that SELDI has lower resolution and lower mass accuracy. In addition, SELDI is unsuitable for high molecular weight proteins (>100 kDa) and is limited to the detection of bound proteins on to the ProteinChip Array. 

 Most studies published about parasitic diseases have focused on SELDI. The SELDI, a derivation of MALDI, allows sample binding to chemically active ProteinChip surfaces. Several types of ProteinChip arrays are available with differing abilities to bind proteins with different chemical (anionic, cationic, hydrophobic, metallic, and normal phase) or biological (antibody, enzymes, receptors) properties, thereby allowing the direct analysis of proteins from complex biological samples without the need for prior separation by 2D gel electrophoresis. The output of the SELDI is a spectrum of mass-to-charge ratios (m:z values) with their corresponding relative intensities (approximating to relative abundance).

 SELDI analyses were initially applied to the discovery of early diagnostic or prognostic biomarkers of cancer (reviewed in [185]). Recently, this technique has been applied to the study of serum biomarkers of infectious diseases such as Severe Acute Respiratory Syndrome [186], African trypanosomiasis [83], fascioliasis [157], cysticercosis [158], and Chagas diseases (Ndao et al., submitted). Such studies have focused on identifying a distinctive configuration of circulating serum proteins that are indicative of a specific pathophysiological state, a so-called “proteomic fingerprint.”

 The real potential of proteomic fingerprinting is in its use as a discovery tool for novel biomarkers that can then be incorporated into simple bedside diagnostics based on affordable technologies such as immunologically based antigen-detection tests that could be implemented in dipstick or cassette formats.

## Figures and Tables

**Figure 1:**
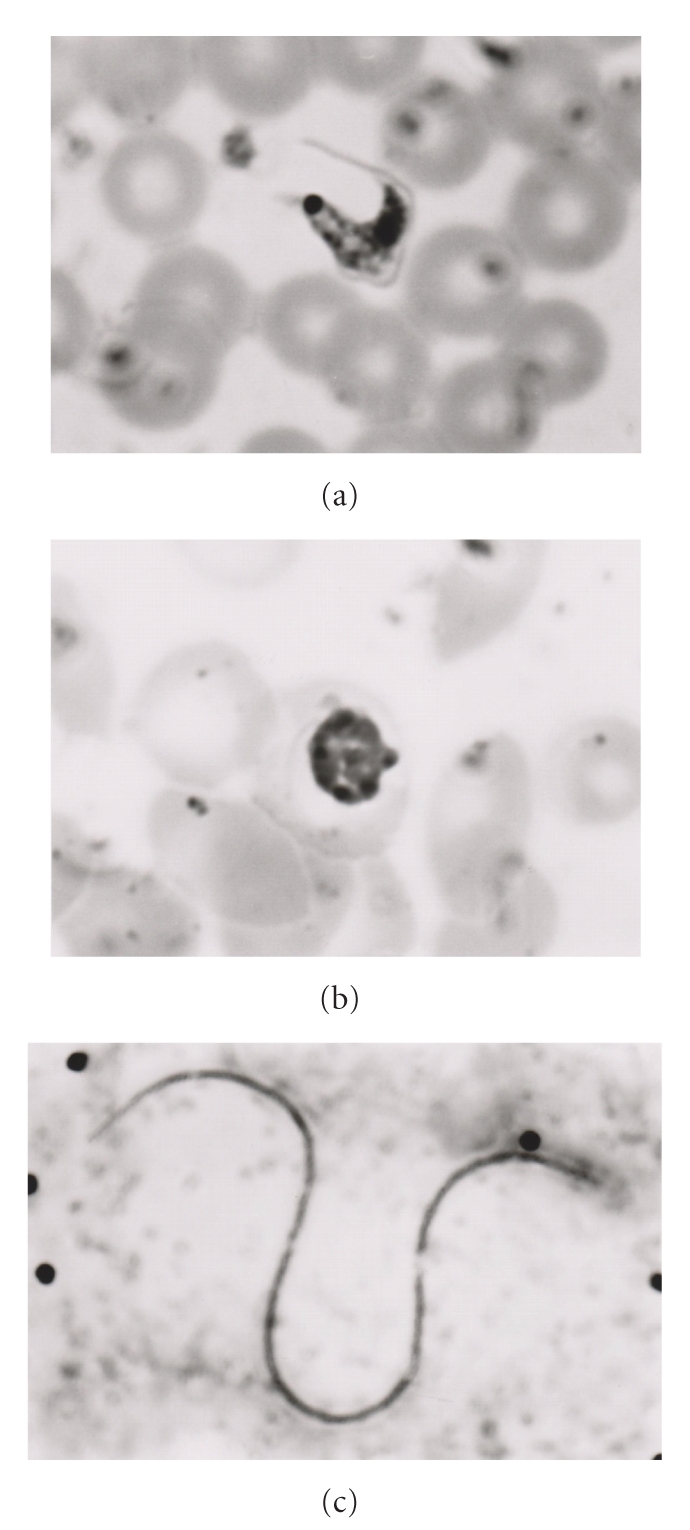
*Microscopy*. Comparison of *Trypanosoma cruzi *trypomastogote (a) with *Plasmodium malariae *schizont (b) and with microfilaria (c: *Mansonella perstans*) in squirrel monkey blood smear. Giemsa stain: 70x oil-immersion objective (a) and (b) and 27.2x objective (c), adapted with permission of Comparative Medicine from [9].

**Figure 2:**
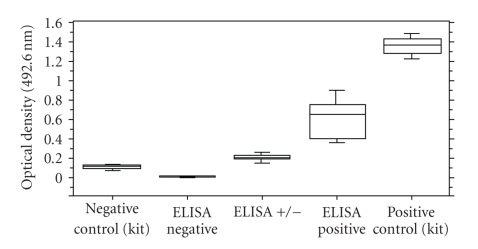
*Serology-based assays: ELISA*. Enzyme-linked immunosorbent assay (ELISA) absorbance values for antibodies to *T. cruzi *in monkey samples. Median values are indicated by horizontal lines within the boxes; the 25th and 75th percentiles are enclosed by the boxes; the 5th and 95th percentiles are enclosed by the bars outside the boxes. Adapted with permission of Comparative Medicine from [9].

**Figure 3:**
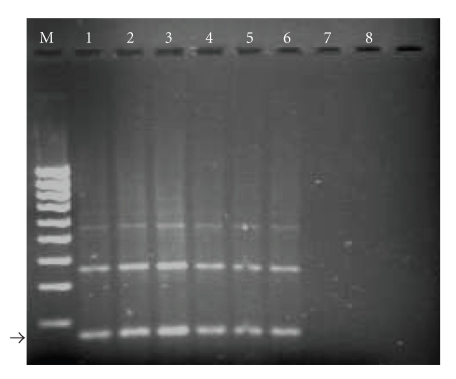
Molecular-based assay: PCR. Example of PCR results obtained for seven monkey samples, using the TCRUZ primers. Blood samples were processed as described in Materials and Methods. The PCR products were electrophoresed in a 2% agarose gel and stained with ethidium bromide. The 168 bp band (arrow) is the expected *T. cruzi*-specific product. The 360 and 550 bp are also specific products resulting from amplification of two or three of the 195 bp repeats found in tandem arrays in the *T. cruzi *genome. Lanes 1 to 6 contain the amplification products of DNA from *T. cruzi-*infected monkeys; 7, blood from noninfected monkey; 8, negative control (distilled water); and M, 100 bp ladder, adapted, with permission of Comparative Medicine from [9].

**Table 1:** Diagnostic tools for the detection of specific blood-borne parasitic diseases.

	African trypanosomiasis	Babesiosis	Chagas disease	Leishmaniasis	Malaria	Toxoplasmosis
	*Trypanosoma brucei*	*Babesia microti*	*Trypanosoma cruzi*	*Leishmania species*	*Plasmodium species*	*Toxoplasma gondii*
MICROSCOPY	[23]	[10]	[11]	[12]	[13]	—

SEROLOGY-BASED ASSAYS	—	—	—	—	—	—

ELISA	[24, 25]	[26]	[27–30]	—	—	[31]
FAST-ELISA	—	—	—	—	[32, 33]	—
Dot-ELISA or Dipstick	[34]	—	[35]	[2, 18]	—	—
RIPA-ELISA	—	—	[36, 37]	—	—	—
DHA or IHA	[38]	—	—	[18]	—	—
DFA or IFA	[39]	[40, 41]	—	—	[42]	[43]
Immunoblot	—	—	[44, 45]	—	—	[46]
PRISM	—	—	[47]	—	—	—
RDT	—	—	[48]	—	[49]	—

MOLECULAR-BASED ASSAYS	—	—	—	—	—	—

PCR	[23]	[50, 51]	[52–54]	[55]	[56]	[57, 58]
RT-PCR	[59]	—	—	[60–62]	[4, 56]	[63]
QT-NASBA	—	—	—	[64]	[65, 66]	—
RT-QB-NASBA	—	—	—	—	[67]	—
LAMP	[68]	—	[69]	—	[70–74]	—
Luminex	—	—	—	—	[75]	—
PCR-ELISA	—	—	—	[62, 76]	[77–79]	—
OC-PCR	[80]	—	—	[81]	—	—

PROTEOMICS	—	—	—	—	—	—

Mass Spectrometry (LDMS, MALDI-TOF, SELDI-TOF)	[82, 83]	—	—	—	[84–86]	—

FAST-ELISA: Falcon assay screening test; RIPA-ELISA: radioimmunoprecipitation assay; DHA or IHA: direct or indirect hemagglutination assay; DFA or IFA: direct or indirect immunofluorescence assay; RDT: rapid diagnostic test; LIPS: luciferase immunoprecipitation system; CATT: Card Agglutination test for Trypanosomiasis; PCR: polymerase chain reaction; RT-PCR: real-time polymerase chain reaction; QT-NASBA: quantitative nucleic acid sequenced-based amplification; RT-QT-NASBA: real-time quantitative nucleic acid sequenced-based amplification; LAMP: loop-mediated isothernal amplification; OC-PCR: oligochromatography Polymerase chain reaction; LDMS: laser desorption mass spectrometry; MALDI-ToF: matrix-assisted laser desorption/ionization time of flight; SELDI-Tof: surface-enhanced laser desorption/ionization time of flight, IFA: immunofluorescent assay, EIA: Enzyme immunoassay, RT-PCR: Real time PCR, IB: immunoblot.

**Table 2:** Diagnostic tools for the detection of specific intestinal parasitic diseases.

	PROTOZOA	TREMATODES		CESTODES		NEMATODES	
	Cryptosporidiosis	Fasciolosis	Schistosomiasis	Taeniasis/ Cysticercosis	Hydatidosis	Filariasis	Strongylodiasis
	*Cryptosporidium parvum, C. hominis*	*Fasciola hepatica, F. gigantica*	*Schistosoma mansoni*	*Taenia solium*	*Echinococcus granulosus, E. multilocularis*	*Wuchereria bancrofti, Brugia malayi, B. timori, Loa loa*	*Strongyloides stercoralis*
MICROSCOPY	[87]	[88]	[89]	—	[90]	[91]	[92]

SEROLOGY BASED ASSAYS	—	—	—	—	—	—	—

ELISA	[93, 94]	[88, 95]	[96, 97]	[98–101]	[102–106]	[14]	[107–111]
FAST-ELISA	—	[112]	[1]	—	—	—	—
Dot-ELISA or Dipstick	—	[113, 114]	[115]	—	[116, 117]	[118–120]	—
DHA or IHA	—	—	[121]	—	[122]	[123]	[124]
DFA or IFA	[93, 125, 126]	—	—	—	—	—	[127, 128]
Immunoblot	—	[112]	[129]	[130, 131]	[122]	—	[132]
LIPS	—	—	—	—	—	[133]	[134]

MOLECULAR-BASED ASSAYS	—	—	—	—	—	—	—

PCR	[135, 136]	—	[137, 138]	[139, 140]	—	[141–143]	[144]
RT-PCR	[145–147]	—	[148, 149]	—	—	—	[150]
LAMP	[151, 152]	—	—	[153]	—	—	—
Luminex	[154]	—	—	—	—	—	—
PCR-ELISA	—	—	—	—	—	[155]	—
OC-PCR	—	—	[156]	—	—	—	—

PROTEOMICS	—	—	—	—	—	—	—

Mass Spectrometry (LDMS, MALDI-TOF, SELDI-TOF)	—	[157]	—	[158]	—	—	—

Abbreviations: see Table 1.

**Table 3:** Diagnostic tools for the detection of specific blood-borne parasitic diseases offered by the CDC and the NRCP.

	African trypanosomiasis	Babesiosis	Chagas disease	Leishmaniasis	Malaria	Toxoplasmosis
	*Trypanosoma brucei species*	*Babesia microti*	*Trypanosoma cruzi*	* Leishmania species*	*Plasmodium species*	*Toxoplasma gondii*
CDC DIAGNOSTIC TOOLS	Microscopy	Microscopy IFA, PCR	Microscopy culture, IFA, EIA	Microscopy, Culture, IFA	Microscopy PCR, IFA	Microscopy IFA, EIA

NRCP DIAGNOSTIC TOOLS	Microscopy, culture, CATT, PCR	Microscopy, IFA	Microscopy, culture, EIA, RT-PCR	Microscopy, Culture, IFA, RT-PCR	Microscopy, IFA, IB, PCR	RT-PCR

CDC: Centre for Disease Control, Atlanta, Georgia, USA. NRCP: National Reference Centre for Parasitology, Montreal General Hospital, Montreal, Quebec, Canada. Abbreviations: see Table 1.

**Table 4:** Diagnostic tools for the detection of specific intestinal parasitic diseases offered by the CDC and the NRCP.

	PROTOZOA	TREMATODES		CESTODES		NEMATODES	
	Cryptosporidiosis	Fasciolosis	Schistosomiasis	Taeniasis Cysticercosis	Hydatidosis	Filariasis	Strongylodiasis
	*Cryptosporidium parvum, C. hominis*	*Fasciola hepatica, F. gigantica*	*Schistosoma mansoni*	*Taenia solium*	*Echinococcus granulosus, E. multilocularis*	*Wuchereria bancrofti, Brugia malayi, B. timori, Loa loa*	*Strongyloides stercoralis*
CDC DIAGNOSTIC TOOLS	Microscopy, EIA, PCR, RT-PCR	—	Microsocopy, FAST-ELISA, IB	Immunoblot,	IB	Microscopy, EIA	Microscopy, EIA

NRCP DIAGNOSTIC TOOLS	Microscopy, EIA	Microscopy, EIA	Microscopy, EIA	IB	EIA	Microscopy, EIA	Microscopy, culture, EIA, IB

CDC: Centre for Disease Control, Atlanta, Georgia, USA. NRCP: National Reference Centre for Parasitology, Montreal General Hospital, Montreal, Quebec, Canada.
